# Les fractures de la tête radiale chez l'enfant: à propos de 66 cas

**DOI:** 10.11604/pamj.2014.17.138.3775

**Published:** 2014-02-27

**Authors:** Karima Atarraf, Mounir Arroud, Lamiae Chater, My Abderrahmane Afifi

**Affiliations:** 1 Service de Traumatologie Orthopédie Pédiatrique, CHU Hassan II, Faculté de Médecine et de Pharmacie, Université sidi Mohammed ben Abdullah, Fès, Maroc

**Keywords:** Fracture, tête radiale, enfant, Fracture, radial head, child

## Abstract

Les fractures de la tête radiale sont des fractures rares, en effet elles représentent 1% des fractures de l'enfant, mais elles restent graves par leurs complications qui sont dominés par les troubles de croissance et surtout la nécrose de la tête radiale. Nous avons pu analyser 66 dossiers chez des enfants âgés de 4 à 14 ans, étude colligée au Service de Traumato-Orthopédie Pédiatrique du CHU Hassan II de Fès, sur une période de 6 ans. l'âge moyen était de 9,4ans. Les fractures stade I et II représente 77.28%. Le traitement orthopédique a été réalisé dans 45 cas. Le traitement chirurgical a été indiqué en 2éme intention chez 21 malades, dont 9 malades ont bénéficié d'une réduction sanglante. L'étude clinique reste pauvre, la radiologie occupe une place prépondérante dans le diagnostic positif et pour l'orientation thérapeutique. Les méthodes sont variables, l'indication et le résultat dépendent du degré du déplacement.

## Introduction

Les fractures de l'extrémité supérieure du radius sont peu fréquentes, elles représentent 1% des fractures de l'enfant, et 4 à 7% des fractures du coude. Le pic de fréquence se situe entre 8 et 13 ans [1]. Elles ont une spécificité pronostique particulière liée probablement à l'importance du déplacement et certainement aux méthodes thérapeutiques. Leur gravité ne doit pas être méconnue compte tenu des séquelles définitives qu'elles peuvent entraîner sur une articulation en pleine croissance et propice à l'enraidissement.

## Méthodes

Nous rapportons une série de 66 cas de fracture radiale, colligée au Service de Traumatologie-Orthopédie Pédiatrique du CHU Hassan II de Fès, sur une période de 6 ans, allant de janvier 2003 à janvier 2013.

## Résultats

**Figure 1:**
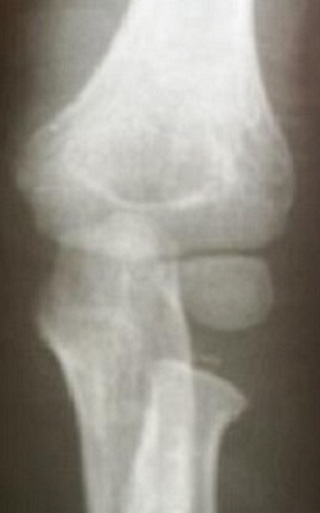
Fracture stade I de Judet

L'âge moyen de nos patients est de 9,4 ans, avec des extrêmes de 4 à 14 ans. 14 de nos patients étaient de sexe féminin. Le coté droit était atteint dans 42 cas (72.72%), Les accidents domestiques restent l'étiologie la plus fréquente des traumatismes. Le mécanisme indirect est incriminé dans 36 cas. La clinique n'est jamais spécifique. Les fractures stade I et stade II représente 77.28% contre 22.7% des fractures stades III et IV. Le traitement orthopédique a été réalisé dans 45 cas dont 41 était classés stade I (Figure 1), avec recours à la réduction dans 4 cas (stade II) (Figure 1). La durée d'immobilisation était de 3 à 4 semaines, assurée par une attelle postérieure. Le traitement chirurgical a été indiqué en 2éme intention chez 21 malades, dont 9 malades (stade III et IV) ont bénéficié d'une réduction sanglante. L'embrochage centro médullaire élastique stable de Métaizeau a été réalisé chez 12 patients, avec poinçonnage percutané dans un cas (Figure 3,Figure 4, Figure 5).

**Figure 2:**
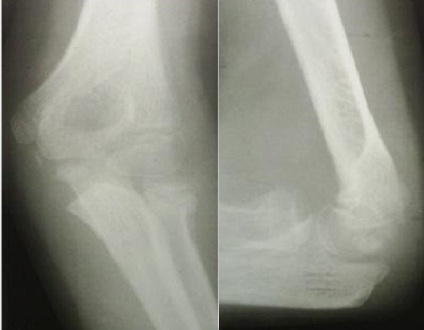
Fracture stade II de Judet traitée orthopédiquement

**Figure 3:**
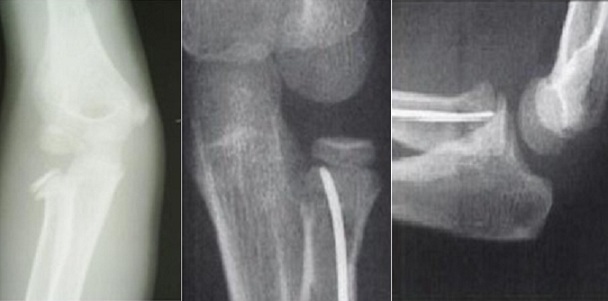
Fracture stade III de Judet traitée par Embrochage Centro-Medullaire Elastique Stable

**Figure 4:**
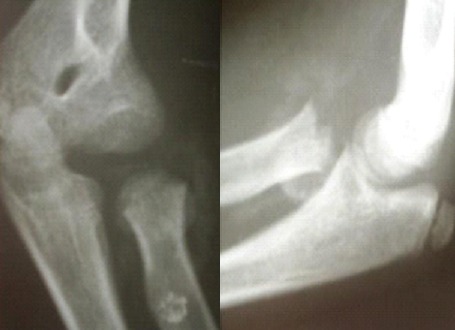
Fracture stade IV de Judet traitée par Embrochage Centro-Medullaire Elastique Stable

**Figure 5:**
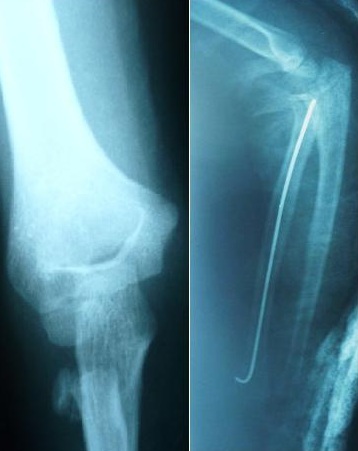
Fracture stade IV de JUDET traitée par Embrochage Centro-Medullaire Elastique Stable

Les résultats ont été appréciés en fonction de la présence ou non d'un gène de la mobilité du coude. Pour cela; nous avons adopté la classification de Steele qui tient compte à la fois de la prono-suppination et de la flexion-extension du coude. Les complications dans notre série ont été dominé essentiellement par la nécrose de la tête radiale et la paralysie de la branche motrice du nerf radial dans un cas chacun ; ce dernier incident a été secondaire au poinçonnage, et a complètement récupéré après rééducation. Les résultats très bon ou bon obtenus avec les méthodes orthopédiques (44.66%) dépassent de loin ceux obtenus par la réduction chirurgicale (33.33%).

## Discussion

Les fractures de la tête radiale constituent une lésion rare chez l'enfant, les caractéristiques épidémiologiques de notre étude sont comparable à celle des autres séries. L'âge moyen de nos patients est de 9,4 ans ; il concorde avec les données de la littérature [2–5], ceci est expliqué par la fragilité importante avant l'ossification complète du cartilage conjugal de l'épiphyse. La nette prédominance masculine rejoint les chiffres publiés dans la série de Tibone (21 garçons) [3]. Pour Robert et Moules [5] ; le coté droit et le coté gauche sont également atteint, contrairement à notre série ou l'atteinte du coté droit prédomine (14 cas) rejoignant la série de Fuentes [5].

Les accidents domestiques restent l'étiologie la plus fréquente des traumatismes [5]. Le mécanisme indirect est incriminé dans 12 cas, ce qui rejoint la série de Robert [5] ou le mécanisme indirect a été en cause chez 25 enfants (25/38). La clinique n'est jamais suffisante pour poser le diagnostic d'une fracture de la tête radiale [4–6], d'ou l'intérêt de la radiologie qui reste un élément primordial pour le diagnostic positif et pour permettre une classification de cette lésion, la plus utilisée reste la classification de Judet. ainsi les fractures stade I et stade II représente 77.28% contre 22.7% de fractures stades III et IV, celle-ci permet également d'orienter la thérapeutique, qui était orthopédique pour les fractures non déplacées dans 11 cas (68.18%) et ayant eu recours à une réduction dans 4 cas (stade II, III, IV). Le traitement orthopédique qui consiste en l'immobilisation par une attelle postérieur brachio-anté brachio-palmaire reste efficace surtout dans les fractures stade I et II [6]. La réduction s'impose dans les grands déplacements ; elle est conseillée au delà d'une bascule de 30° afin de ne pas compromettre les mouvements de la prono-suppination [1]. Même si la réduction orthopédique reste possible, celle-ci est souvent rendue difficile car elle s'applique sur un coude le plus souvent oedematié. La durée d'immobilisation est de 3 à 4 semaines.

La rareté des fractures du col radial chez l'enfant et la bonne tolérance fonctionnelle des déplacements modérés expliquent la place assez restreinte du traitement chirurgical de ces lésions. Ainsi dans notre série il a été indiqué en 2eme intention chez 7 malades soit 31.82%, dont 3 malades (stade III et IV) ont bénéficié d'une chirurgie sanglante, une telle réduction est actuellement à éviter car elle compromet sérieusement la vascularisation épiphysaire déjà menacée par l'importance du déplacement. Une telle attitude agressive peut entrainer une nécrose de la tête radiale avec stérilisation du cartilage de croissance.

L'Embrochage Centro-Medullaire Elastique Stable (ECMES) a été indiqué dans les stades II et III chez 03 malades, Metaizeau rapporte en 1988, 12 cas d'enfants âgés de 8 à 13 ans traités par cette méthodes dont le déplacement était compris entre 30 et 75% [7]. Une telle technique a comme avantages de réaliser une réduction du foyer fracturaire à l'aide d'une broche sans abord du foyer fracturaire, ce qui diminue le risque de la nécrose de la tête en plus du respect de l'hématome fracturaire. Le poinçonnage percutané prônée depuis une quinzaine d'années par R.KOHLER représente un compromis entre la réduction orthopédique qui est souvent difficile; voir impossible dans les stades III et IV et l'abord chirurgical dont les conséquences fonctionnelles sont plus importantes que les bénéfices obtenus, le poinçonnage percutané a été réalisé chez un seul malade dans notre série.

La raideur du coude est une complication qui reste fréquente mais le plus souvent palliée par la rééducation active. Un cas de paralysie de la branche motrice du nerf radial a été noté dans notre série avec une récupération totale après rééducation. Nous n'avons constaté aucun déplacement secondaire, ni trouble trophique. On a déploré un seul cas de nécrose céphalique, dont l'évolution a été marquée par la raideur du coude. Les résultats très bon ou bon obtenus avec les méthodes orthopédiques (44.66%) dépassent de loin ceux obtenus par la réduction chirurgicale (33.33%). Ceci ne diminue en rien de la valeur du traitement chirurgical mais au contraire car les résultats excellents observés avec le traitement orthopédique sont du à la fréquence des stades I qui en général sont des très bons pronostics.

Dans notre série l'immobilisation plâtrée seule donne de très bons résultats dans 66.66% des cas, et de bons résultats dans 33.33%. Ce qui rejoint celui de VONKLOES [8]. Les résultats du traitement chirurgical était satisfaisant dans 71.4% et mauvais dans 14.28% (1cas traité /foyer ouvert). Robert [5] conclut dans son observation que les résultats imparfaits sont plus fréquents dans les cas opérés. Pour Steele et Graham [9]; les résultats sont trop satisfaisant dans 33 cas sur 36 avec la technique de Métaizeau.

## Conclusion

Les fractures de la tête radiale chez l'enfant sont rares, de diagnostic essentiellement radiologique, leur pronostic est étroitement lié à la méthode thérapeutique. Ainsi la réduction orthopédique doit avoir les indications les plus larges possibles, et la chirurgie doit se contenter d'un résultat même anatomiquement imparfait, laissant un maximum de chance de récupération fonctionnelle grâce au remodelage métaphysaire [3].
